# The Effect of Therapeutic Approaches on Hallux Valgus Deformity

**DOI:** 10.7759/cureus.58750

**Published:** 2024-04-22

**Authors:** Alfiza Z Khan, Deepali S Patil

**Affiliations:** 1 Department of Musculoskeletal Physiotherapy, Ravi Nair Physiotherapy College, Datta Meghe Institute of Higher Education and Research, Wardha, IND

**Keywords:** soft tissue mobilization, joint kinematics, physical therapy, flexibility, hallux valgus

## Abstract

Hallux valgus (HV) is a relatively frequent disease caused by a complicated structural malformation of the primary ray. The bunion or middle projection generated by the hallux's lateral displacement and pronation is merely one element of the three-dimensional abnormality. HV may trigger severe discomfort and affect joint kinematics. The specific kinematic cause is still unknown. Female age, gender, restrictive footwear, and heritage are risk indicators. HV frequently coexists along metatarsal adducts, equines contracture, hammertoe imperfection, and pes planus. HV is a frequent foot ailment with multiple, complicated, unknown etiology and course. HV has a preference for females. It is an ongoing condition for which there is no known treatment to reduce or prevent improvement. Fibrodysplasia ossificans progressiva (FOP) is distinguished by hereditary symmetrical HV deformities or symptoms that begin heterotopic calcification that is either idiopathic or caused by trauma, such as subcutaneous immunizations. Localized heterotopic calcification may be preceded by aggravating, recurring soft-tissue enlargements (flare-ups). Heterotopic calcification may happen anywhere; however, it most commonly impacts locations near the axial bone structure during the early/mild phases until advancing to the appendicular skeleton. As an effect of calcification affecting the flexibility of the joints, it might cause limitations in motion. The initial line of therapy focuses on non-surgical methods including night splinting, orthotics, and larger shoes. The next suggested line of action is surgical intervention if conservative therapy fails. Patients have good postoperative tolerance, and bone union often happens six to seven weeks after surgery. Stretching exercises help to restore function by extending shortened soft tissue and restoring range of motion (ROM). The goal of joint mobilization, a form of manual treatment method, is to extend the ligament, the soft tissue surrounding the limited joint, and the restricting joint capsule by applying modest amplitude passive movement to the joint components.

## Introduction and background

Hallux valgus (HV) is the most prevalent metatarsal imperfections that can cause inflammation and limited movement. The progression of HV is a complex approach. Various inherent outside factors take responsibility [1]. The origins, along with the evolution of HV, are unknown. It is multifaceted, complicated, and has little understanding. HV has a preference for females. It is an incurable illness for which there is no known treatment to decrease or restrict improvement. When nonoperative measures fail in fit individuals, surgery is required. Several techniques are being documented, including tissue repair and bone restoration of the initial ray. The method recommended is determined by the degree of severity of the abnormality [2]. A frequently occurring exception of the metatarsal along with toes is HV. Because of the apparent appearance, HV affects 23% of 18 to 65-year-olds and 35% of people older than 65 years. This is also referred to as a ganglia and bunion. In optimum posture, the largest foot shifts toward the outside part of the foot. Each initial ray contains an axial pattern that is naturally unsteady or is stabilized by a complex interplay among stationary (capsule, ligaments, and plantar fascia) or kinetic (peroneus longus and smaller feet muscular) stabilizers. This muscle's equilibrium may be thrown off at certain feet by a hereditary propensity for irregular osseous alignment and flexibility among the static stabilizers. This procedure might be sped wearing inappropriate footwear, although job, heavy walking, or weight bearing are uncommon for making a difference, thereby enabling treatment (conservative or surgical) to be tailored to the individual [3]. An uncommon autosomal dominant condition characterized by the progression of extraskeletal ossifying and skeletal abnormalities is called fibrodysplasia ossificans progressiva (FOP) [4].

Knowing the strength and trajectory of the stresses exerted upon the forefoot's particularly stress-laden tissues is crucial, especially if surgical intervention is being considered. Recognizing the route mechanics of some foot problems can also be useful. However, such information is not currently accessible [5]. A pair of muscle control strategies are used for assessing extrinsic toe flexor factors or immediate tendon activities. These techniques include an isometric displacement oversight, which has hypothetical myotendinous junctions to an unchanged position, and force feedback management, which corresponds to tendons required to be estimated from normal electromyographic patterns [6]. In the conclusion of the positioning stage of motion in regular extrinsic muscle contractions and an intact plantar fascia, in the absence of extrinsic toe flexor activity (no flexor hallucis longus or flexor digitorum longus) with an intact plantar fascia, and following accomplish fasciotomy in regular extrinsic toe flexor activity, the amount of plantar pressure beneath the foot is determined [7]. Lower limb muscles must put in much effort during an ordinary person's walking motion, particularly during the stance period. Consequently, the main goal of gait analyses is to record electromyography (EMG) activity from various lower limb musculature. The goal for both surfaces and intramuscular EMG techniques is to study the time and magnitude of muscle stimulation [8]. According to the latest pedobarographic assessments, there is reduced movement in the big toe and the initial ray following HV surgery, although satisfactory clinical and radiographic outcomes [9]. Two different ideas explain whether central and transmitted metatarsalgia develops. Initially, the metatarsal experiences dynamic compression while HV intensity grows. Secondly, it is suggested that longer rather than shorter metatarsals can cause metatarsalgia. While treating primary ray degenerative conditions, such as HV or hallux rigidus, it is essential to prevent overly shortening the initial ray. There are conventional and operative approaches for treating metatarsalgia after an unsuccessful HV repair [10].

With regards to HV, prevention and treatment of HV can be operating or preventative. Different forms of care have been used including rehabilitation, surgery, and medicinal or gentle therapy. Other operations including proximal versus distal metatarsal osteotomies, tarsometatarsal (TMT) arthrodesis, and repositioning with lesser rays are used for managing the ailment [11]. Cushions or foot straighteners help relieve inflammation from little digits. Broad, smooth footwear is beneficial to leave adequate space for the feet. However, an operation is required when mallet and clawed feet appear [12]. Considering specialized athletic endeavors like dance, ballet, or gymnastics typically start at a young age and anomalies in development might interact with instruction, it is essential to evaluate hallux deformities in teenage groups [13]. Conventional measures for treating HV include physical therapy, occupational therapy, orthoses, insoles, kinesiology taping, activities, and surgery [14]. Kinesiology taping employs the kinetic effect of an elasticity adhesive tape on the musculoskeletal system as a form of rehabilitation. The kinesiology tape is stretched from 30-40% to create the mechanical effect of hallux abducted; hence, it is believed that the tape's reduced flexibility during balancing taping prevents the hallux from deviating towards the second toe [15]. Additionally, there is a non-invasive medical equipment called the Archercise biofeedback approach, which is made to help people exercise their foot musculature correctly. By employing a footplate sensor to detect changes in bladder pressure brought on by foot arch movement and position, the Archercise gadget provides matching real-time biofeedback via a visual display that actively directs the user's foot through a number of foot training exercises [16]. For healthy individuals without foot diseases, the Archercise biofeedback device seems to be a practical and safe way to help with foot-doming exercises and increase adherence to proper technique.

## Review

Methodology

**Figure 1:**
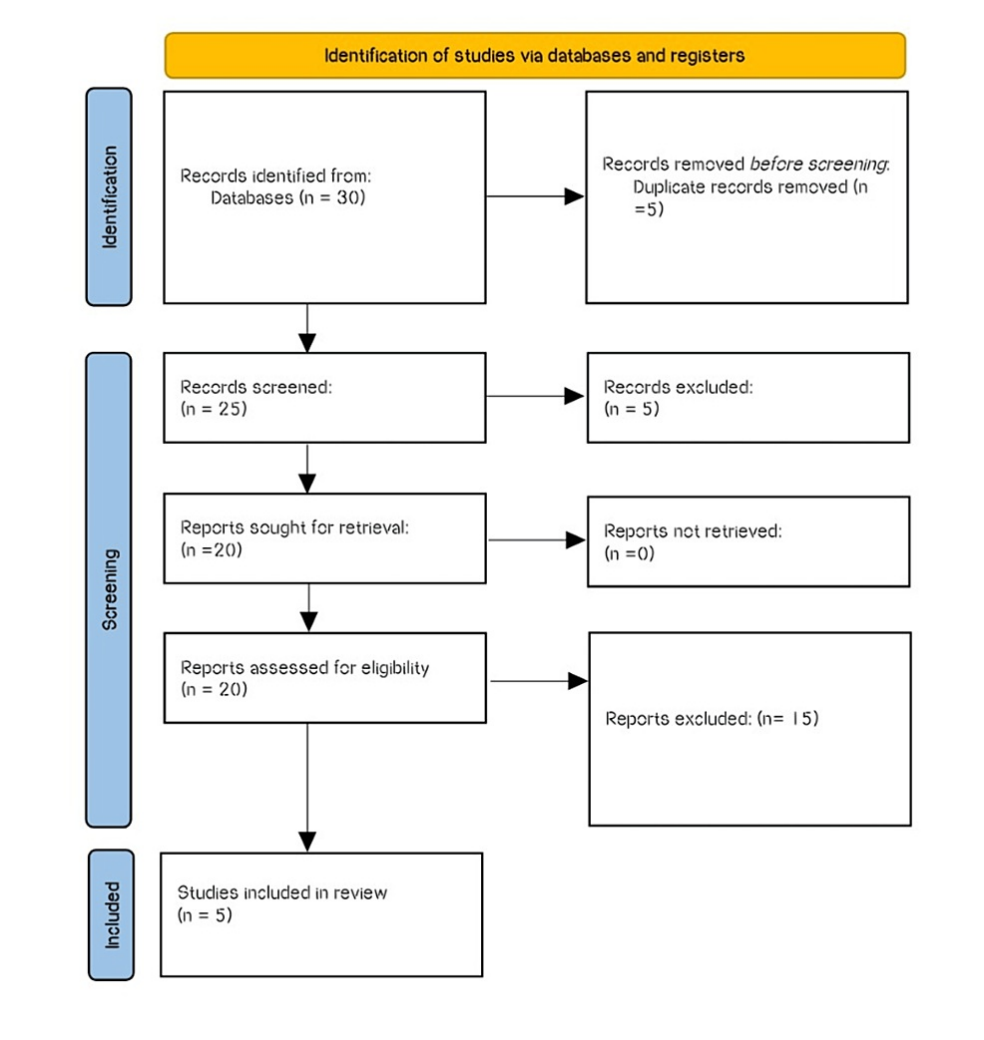
The Preferred Reporting Items for Systematic Reviews and Meta-Analysis (PRISMA) flowchart

Papers from 2009 to 2022 were discovered in databases like Web of Science, Scopus, Google Scholar, and PubMed. The keywords used were: "joint kinematics", "physical therapy", "flexibility", "soft tissue mobilization", and "hallux valgus". Studies that were experimental trials were included. The research papers were chosen based on the criteria of experimental studies, physiotherapy, and rehabilitation with a focus on gait training, and articles should have been published between 2009 and 2022. The study did not include case series, cohorts, reviews, and non-experimental studies. Based on the criteria for inclusion, five articles that specifically emphasized stretching, gait pattern, and soft tissue mobilization in the case of HV were selected after the preliminary screening of 15 pieces. The Preferred Reporting Items for Systematic Reviews and Meta-Analysis (PRISMA) flowchart is illustrated in Figure 1.

Rehabilitation sessions

Session 1

Session 1 was provided to alleviate edema, lymphedema, and venous edema. The foot was elevated, lymph drainage was performed, muscular pump was activated, and cryogenic treatment (cold compresses) was employed [5]. Plantar dorsi flexibility of the foot, along with flexion and extension of the hip and knee, are examples of muscular pump activity. Scar tissue massaging and cold therapy were given over both feet and initial metatarsophalangeal (MTP) articulation areas using cold and warm packages. Each MTP segment underwent mobilization. These alterations were made to enhance the dorsiflexion of the primary MTP bone and plantar flexion within MTP tendons two through five [11]. These comprised terminal phalanx caudal sliding to enhance plantar flexion and proximal phalanx dorsal sliding to enhance dorsiflexion. Moving pressure was a manual therapeutic intervention that activated the mechanoreceptors, which suppress afferent sensations of pain. MTP bones were the primary objective of this rehabilitation method [10]. In an attempt to support the metatarsus, a proximal hand was set in the MTP joint range. Then, the distal hand was developed over the phalanx closest to the joint. For gait training, one of the goals in the session's walking conditioning was achieving the benefit of possible distribution of strain across every aspect of the foot throughout every part of the stance timing, emphasizing starting at the MTP articulation while engaging the hallux [14].

Session 2

Mobilization that occurred during session 2 was comparable to in session 1. Furthermore, the heel bone became stable when the big toe was pronated. Additionally, the rehabilitation therapy carried out individual navicular and cuneiform caudal slides towards the talus and navicular, respectively [10]. The patient was positioned supine during this procedure. When the proximal hand was applied longitudinally, stress concentrated upon the bone, and the distal palm of the physical therapist supported the plantar features of the manipulated areas. Such mobilization movements can be carried out while sitting down [9]. Soft-tissue strategies were employed to enhance the circulation while minimizing stress within the flexor hallucis longus, adductor hallucis (oblique and transverse heads), abductor hallucis, and tibialis anterior musculature. Additionally, from the supine and seated positions, soft-tissue methods were used on peroneal fascial loge in conjunction with active pronation [12].

Motor learning/strengthening exercises*: *HV can be a sign of muscular imbalances caused by adductor hallucis with abductor hallucis. A toes-spread-out (TSO) activity can be advised for the prevention of HV malformation. The lumbrical, interosseous, adductor hallucis, peroneal longus, brevis and flexor hallucis longus, and brevis muscles are the targets for these activities. Currently, active pronation has become a part of this workout [14]. The action can be done using Theraband flexible bands to build endurance. Clients initially perform the activities in a vigorous, enthusiastic manner. Movements of development occur following eccentric therapy. To accomplish it, marble-picking and concentrated training activities of the biggest toe flexors with extensors had to be carried out.

Exercise of gait: When standing, the necessary muscular control of the forefoot's transverse arches was developed at both the mid-stance and terminal stance positions. The active involvement in push-off (barefoot) was the primary objective [14].

Session 3

Sensmotoric training: This therapy mode aimed to improve intramuscular synchronization. Most of the activities were carried out at a standing (mid-stance) posture, gradually increasing weight exerted on the weight-bearing foot before switching to a one-foot stance while maintaining an upright, long-standing leg axis. The eyelids were raised and blocked to concentrate on the neuromuscular feedback mechanism (proprioceptors) [4].

Gait training: Increased intensive pronation, weight bearing on the initial MTP joint, stress carrying, and push-off of the hallux were the main goals of gait training. Clients were to perform a calf enhancement to full weight-bearing upon the big toe, maintaining the appropriate leg axis and climbing stairs, using a concentration to maintain regulation on the knee in a neutral position as well as get enough control of the pelvic region regarding stress towards abduction and external rotation that appears to be the typical weakened structure. Initial findings indicate that a strong rectification may be sustained using the modified Lapidus treatment approach, which can be applied in a range of HV situations and severity [17].

Surgical intervention

Prior to implementing any decisions, it is critical to comprehend the patient's demands. It is important to remember that treating the patient's genuine ailments should come first rather than just making cosmetic corrections [18]. For the decision‐making of individualized surgical strategies, such as to improve the therapeutic impact of HV surgery, the distal soft tissue operation is considered a useful supplemental surgical procedure. This delicate tissue procedure aims to restore the physiological balance of capsular, ligamentous, and muscular structures around the first MTP joint [19]. The significant incidence of recurrent episodes in the management of substantial teenage congruence HV malformations led to the development of the double first metatarsal osteotomy procedure. The HV malformation can be effectively corrected by a double first metatarsal osteotomy (basal opening wedge and distal chevron osteotomy) combined with Akin osteotomy [20]. Among the best-known distal metatarsal osteotomies is Mitchell's process, which entails a double step-cut osteotomy at the level of the first metatarsal neck, initially proposed by Hawkins in 1945. Since the procedure's original introduction, several changes have been suggested to avoid the difficulties that have been associated with it [21]. Upon the dorsal face of the metatarsal base, the Ludloff osteotomy is an oblique osteotomy that begins 1.5 cm distal to the metatarsal cuneiform articulation [22]. An operation with a scarf osteotomy is performed in moderate to severe HV abnormalities [20].

A superficial incision can reveal the initial foot ray metatarsal bones on the inside of the feet. This is cut in Z-pattern during an osteotomy (repositing the newly formed bones) and then fastened in a new direction utilizing tiny screws. The preferred course of treatment for the initial phases is cheilectomy. Following full weight-bearing within a postoperative shoe having a straight, firm sole that is used for six weeks, most forefoot surgeries occur [22]. Clients are encouraged to elevate the affected foot for most of the initial two weeks because of its recurrent propensity for inflammation. Within eight to 12 weeks, clients may generally walk freely. Implants may be eliminated six to nine months following an operation, but if they are not causing any complaints in senior individuals, they can be left in place [12].

A study by Kyeong-Ah Moon (2022) looked into how quickly a manual stretching technique impacted HV individuals. The research showed that in participants with mild HV, manual stretching maneuver (MSM) can help reduce the HV angle. Muscle imbalance results from more extreme HV. Physical treatment calls for a mix of stretching and strengthening exercises to avoid HV. Thus, the verdict is that MSM can reduce the HV angle in people with mild HV. However, additional longitudinal clinical investigations are necessary to examine the long-term impacts of MSM in individuals with HV [23].

Research done by Jesse Steadman and Charles L. Saltzman (2021) demonstrated the HV deformity's first metatarsal rotation in the early descriptions of the pathoanatomic of the HV deformity. Rotation of the first metatarsal (M1) was identified as a probable etiological component. However, because biplanar radiographs have traditionally been the preferred approach for visualizing HV, physicians primarily focused on developing measuring techniques and correction procedures limited to two dimensions, medial-lateral and inferior-superior [24].

Sheree E. Hurn (2022) demonstrated that the inclusion of foot activities along with orthoses, splinting, manipulation, and tape did not significantly affect the major objectives for a variety of a meta-analysis. Yet, using foot orthoses, nocturnal splinting, dynamic splinting, manipulation, and strapping coupled with foot movements, a multimodal physiotherapy program, or botox injections significantly reduced inflammation, according to outcomes of eight research. Four trials found that using sleep splinting, foot activities, comprehensive physiotherapy, or botox injections reduced the HV angle by a clinically relevant amount [25].

Saba Sadra (2013) showed that HV is an independent risk factor for falls in older individuals and is connected to worse performance in gait and balance activities. The authors set out to determine whether corrective HV surgery enhances balance and gait. Around 40 adults' speed and static balancing data were collected using a cross-sectional study methodology. This study offers evidence suggesting that corrective lower-extremity surgery can improve certain aspects of balance control [26]. Przemysław Lisiński (2015) showed that it was challenging to compare the trials because they used varied therapies and distinct approaches. Nevertheless, the entirety of the investigations noted positive outcomes following the application of different rehabilitation techniques. The more frequent advantages include pain alleviation or deformities repair [27].

Discussion

The effectiveness of nonsurgical therapies for HV is not well established, however, a decrease in pain seems more likely than an increase in HV angle [25]. In the present research, individuals who experienced HV operations and subsequent rehabilitation or exercise for gait had their plantar pressure distribution measured. During the initial or final evaluation at six months following a procedure, the stress variables in the main toe area or the area surrounding the initial metatarsal bone did not generally reduce. Following the procedure for HV, multiple researchers examined alterations to plantar stress distribution and discovered lower stress characteristics via the hallux with initially metatarsal head regions. To the extent of our information, these analyses of after-surgical protocols included neither rehabilitation nor locomotor education. Still, they came to a finding because doing so would benefit the therapeutic result [28]. An HV was linked to joint range of motion (ROM) and lower extremity alignment. Prospective studies might pinpoint anatomical indicators of risk, nonsurgical preventive strategies, and surgical therapeutic adjuncts for the development of HV [29]. The initial hypothesis that HV has a detrimental impact on spatiotemporal metrics and foot kinematics while walking as well as on knee, hip, and pelvic kinematics in comparison to competent controls was validated by the data [30]. The majority of cases of HV deformities in women are attributed to high heels and small or tight shoes. Prior research has concentrated on orthoses, taping, footwear changes, and surgery therapy; however, these methods do not address valgus deformities and might lead to specific problems [31]. In the past few years, arthroscopic has been used increasingly often for managing first MTP joints. Although gout, osteochondritis dissecans, and HV have all been treated with it, hallux rigidus is the most typical symptom [32].

Along with relieving inflammation while improving lymphatic circulation, the flexible rehabilitative bandages utilized in the present research have been found to rectify pelvic or shoulder misalignments. While the approach and length of taping administration varied in the studies, it was noted that during 23 episodes of employing kinesiology tape in a patient with mild HV, the HV angles had significantly decreased, as evidenced via X-ray pictures. Walking on the beach's sand or treating ultrasonic frequencies and intensity with copper sulfate solution works together for enhanced outcomes [33]. The operation on HV aims to repair a defect by rebalancing the initial ray and the initial MTP joints morphologically while functioning. Distal metatarsal osteotomies or various forms offer many successful clinical or radiological results. The operative strategy, bones sliced, or anchoring mechanism varies between procedures. Among the more popular treatments for low to severe HV involve a chevron osteotomy, in which various publications report positive medical outcomes. Clients with HV who receive physical therapy benefit from this immediately following surgery and if it is given as a primary therapeutic. Though rehabilitation fails to fix deformations, it may ease discomfort, enhance gait performance, or increase footwear comfort. For this reason, individuals with HV should be evaluated for rehabilitation in addition to surgery [27]. According to this study's findings, the plantar pressure characteristics shift from the medial to the lateral portion of the forefoot as one increases HV inclination. In addition, the sesamoid complex's location, which modifies its pressure parameters as it subluxates laterally and shifts the load, must be taken into consideration [34]. For patients with mild to severe symptomatic HV, dry needling is a useful and efficient technique for achieving better first MTP joint realignment.

## Conclusions

During HV operation, various authors have noted unusual patterns of gait. Across individuals who underwent HV operations followed by rehabilitation and locomotor training in aftercare, we observed an improvement in plantar pressure variables in the area of the great toe with the initial ray. As a result, we think that postoperative physical therapy aids when regaining the capacity for walking with weight-bearing following the HV operation. Suffering under bunion (HV) may be difficult and aggravating. Therapeutic exercise is an effective approach for strengthening the tissues surrounding the bunion. It also alleviates inflammation and expands movement while enhancing equilibrium. Deformities cannot be corrected nonoperatively. Yet, using orthopedic shoes or rehabilitation in addition to orthotics might reduce the discomfort. Subsequently, different physiotherapist modalities can be used during a single healing phase and when different treatment modalities merge (for instance, soft tissue mobilization plus stationary stretches). Surgical is recommended once a hammer toe is created. Therefore, it is necessary to undergo physical rehabilitation after that for better gait form while maintaining an efficient ROM. Regular kinesiology tape-balanced taping might be an additional therapy for mild HV. According to research, stretching and mobilization techniques show greater positive benefits than toe spreading and brief foot workouts for repairing deformities. Patients with HV benefit from physical therapy when it is administered as a fundamental treatment as well as during the postoperative phase. Even in cases when deformity cannot be corrected, physiotherapy can nevertheless alleviate discomfort, enhance gait, and make wearing footwear more comfortable. Effective in improving HV, soft tissue mobilization needs standard follow-up and daily management. Static stretching application significantly reduces pain, decreases musculotendinous stiffness, and relaxation elongation with least follow-up. In feet with HV, several inherited or acquired biomechanical anomalies have been found. These correlations are nonlinear and imperfect though. Any patient’s HV is the result of several contributing causes. A more rational strategy for managing HV will be feasible if this intricate etiology is recognized, allowing for individualized therapy (conservative or surgical).
